# IL4I1 in M2-like macrophage promotes glioma progression and is a promising target for immunotherapy

**DOI:** 10.3389/fimmu.2023.1338244

**Published:** 2024-01-05

**Authors:** Feng Ye, Lichong Wang, Yuanyou Li, Chengyuan Dong, Liangxue Zhou, Jianguo Xu

**Affiliations:** Department of Neurosurgery, West China Hospital, Sichuan University, Chengdu, China

**Keywords:** IL4I1, glioma, tumor microenvironment, immunotherapy target, M2-like macrophage, TAM, biomarker

## Abstract

**Background:**

Glioma is the prevailing malignant intracranial tumor, characterized by an abundance of macrophages. Specifically, the infiltrating macrophages often display the M2 subtype and are known as tumor-associated macrophages (TAMs). They have a critical role in promoting the oncogenic properties of tumor cells. Interleukin-4-induced-1 (IL4I1) functions as an L-phenylalanine oxidase, playing a key part in regulating immune responses and the progression of various tumors. However, there is limited understanding of the IL4I1-mediated cross-talk function between TAMs and glioma cell in the glioma microenvironment.

**Methods:**

TCGA, GTEx, and HPA databases were applied to assess the IL4I1 expression, clinical characteristics, and prognostic value of pan-cancer. The link between IL4I1 levels and the prognosis, methylation, and immune checkpoints (ICs) in gliomas were explored through Kaplan–Meier curve, Cox regression, and Spearman correlation analyses. The IL4I1 levels and their distribution were investigated by single-cell analysis and the TIMER 2 database. Additionally, validation of IL4I1 expression was performed by WB, RT-qPCR, IHC, and IF. Co-culture models between glioma cells and M2-like macrophages were used to explore the IL4I1-mediated effects on tumor growth, invasion, and migration of glioma cells. Moreover, the function of IL4I1 on macrophage polarization was evaluated by ELISA, RT-qPCR, WB, and siRNA transfection.

**Results:**

Both transcriptome and protein levels of IL4I1 were increased obviously in various tumor types, and correlated with a dismal prognosis. Specifically, IL4I1 was implicated in aggressive progression and a dismal prognosis for patients with glioma. A negative association was noticed between the glioma grade and DNA promoter methylation of IL4I1. Enrichment analyses in glioma patients suggested that IL4I1 was linked to cytokine and immune responses, and was positively correlated with ICs. Single-cell analysis, molecular experiments, and *in vitro* assays showed that IL4I1 was significantly expressed in TAMs. Importantly, co-culture models proved that IL4I1 significantly promoted the invasion and migration of glioma cells, and induced the polarization of M2-like macrophages.

**Conclusion:**

IL4I1 could be a promising immunotherapy target for selective modulation of TAMs and stands as a novel macrophage-related prognostic biomarker in glioma.

## 1 Introduction

Gliomas are classified into low-grade gliomas (LGG) and glioblastoma (GBM), which are the most common intracranial tumors of the adult central nervous system, with significant heterogeneity and adaptability. Despite interventions (e.g., precise resection aided by a neuro-navigation system, post-operative radiotherapy, and concurrent temozolomide chemotherapy), the median survival for glioma patients is ≤ 15 months, with a 5-year survival of ≤10% (1–4). Thus, a deeper understanding of the tumor microenvironment (TME) and immunologic features of gliomas is needed to offer novel and valuable approaches for a clinical diagnosis and treatment in individuals with glioma.

The TME plays a pivotal part in tumor behavior because it governs the progression and metastasis of tumor cells, and resistance to adjuvant anti-cancer drugs (5–8). Unlike most peripheral tumors, glioma is identified by a significantly suppressive and distinctive “cold TME,” with a paucity of infiltration by T cells but abundant macrophage infiltration (9). Macrophages comprise approximately 30%–50% of gliomas (10). Among them, 15% originate from endogenous microglia and the remaining 85% are recruited from monocytes through tumor-derived cytokines (11, 12). Moreover, macrophages are typically categorized into two different types: M1 (which are pro-inflammatory) and M2 (which are immunosuppressive) (13). Within the TME, infiltrating macrophages often exhibit the M2 phenotype and are referred to as TAMs (14). Multiple studies have shown that TAMs are linked to glioma progression, including tumor proliferation, tumor metastasis, and evasion of the immune system, which leads to higher tumor grade and a worse prognosis (14–16). Therefore, exploring the mechanism by which TAM drives glioma progression could promote the development of novel and attractive immunotherapeutic target against glioma.

IL4I1 could catalyze the conversion of tryptophan (Trp) to indole-3-pyruvic acid (I3P), thereby influencing various cellular processes and immune properties (17). Recently, IL4I1 has been recognized as a promising target for cancer treatment (18). Single-cell analysis showed that IL4I1 promotes the development of ovarian cancer (OV) and is a critical gene in the Riskscore (19). Some researchers have found that IL4I1, as a prognostic biomarker, augments tumor growth by reprogramming TME in melanoma, kidney renal clear cell carcinoma (KIRC), and liver hepatocellular carcinoma (LIHC), which is attributed to its association with the movement and recruitment of leukocytes (20–23). Furthermore, IL4I1 expression has been detected in mesenchymal stromal cells of head–neck squamous cell carcinoma (HNSC) and contributes to immune response modulation and T-cell suppression (24). Specifically, IL4I1 limits the function of CD8^+^T cells and expedites the progression of chronic lymphocytic leukemia (25–27). Those findings underscore the key part of IL4I1 in immune system regulation and tumor progression. Nonetheless, there are few systematic and comprehensive studies about the role of IL4I1, especially in the connection between TAMs and the glioma microenvironment.

In this research, we wish to identify the importance of IL4I1 and its potential as a prognostic and immunotherapeutic biomarker in glioma. For the first time, we revealed the patterns of IL4I1 expression and its role across 33 tumor types, focusing on the difference in both mRNA and protein expression within normal and tumor tissues, and evaluated prognostic significance of IL4I1 expression. With a specific focus on glioma, we delved into the correlation between IL4I1 expression and the malignant progression of glioma by multiple forms of bioinformatics analysis: prognostic value, malignant progression, DNA methylation, single-cell transcriptome, and immune functions. Lastly, we showed an IL4I1-mediated cross-talk mechanism between TAMs and glioma cells, as well as its role in macrophage polarization, through a combination of diverse cellular and molecular experiments: co-culture, Western blotting (WB), quantitative real-time PCR (RT-qPCR), immunohistochemistry (IHC), immunofluorescence (IF), enzyme-linked immunosorbent assay (ELISA), and siRNA transfection.

## 2 Materials and methods

### 2.1 Data acquisition

From TCGA by using UCSC Xena, mRNA-seq and the corresponding clinical information of the pan-cancer dataset (containing 11,006 samples across 33 different cancer) were acquired (https://xena.ucsc.edu/) (28). Additionally, mRNA-seq and clinical data for glioma patients were retrieved from Gliovis database [GlioVis - Visualization Tools for Glioma Datasets (cnio.es)], including three independent datasets (CGGA, Rembrandt, and GSE16011, **Table 1**) (29). The downloaded data were Log2-transformed, and *t*-tests were conducted on tumor and normal tissues; *p* < 0.05 was considered statistically significant. Details for the 33 cancers are listed in **Table 2**.

**Table 1:** Three independent datasets used in this study.

Dataset	Data type	WHO grade II	WHO grade III	WHO grade IV
CGGA	RNA-seq	291	334	388
Rembrandt	Microarray	98	85	130
GSE16011	Microarray	24	85	159

**Table 2:** The details of 33 cancers in TCGA and GEPIA.

Cancer type	Full name	TCGA			GEPIA		
		Tumors	Normal	*p*-value	Tumors	Normal	*p*-value
ACC	Adrenocortical carcinoma	79	–	–	77	128	–
BLCA	Bladder urothelial carcinoma	408	19	**	404	28	*
BRCA	Breast invasive carcinoma	1,098	113	***	1,085	291	*
CESC	Cervical squamous cell carcinoma	306	3	–	306	13	*
CHOL	Cholangiocarcinoma	36	9	***	36	9	*
COAD	Colon adenocarcinoma	458	41	***	275	349	*
DLBC	Diffuse large B-cell lymphoma	48	–	–	47	337	*
ESCA	Esophageal carcinoma	162	11	***	182	286	*
GBM	Glioblastoma multiforme	167	5	**	163	207	*
HNSC	Head and neck squamous cell carcinoma	502	44	***	519	44	*
KICH	Kidney chromophobe	65	24	**	66	53	–
KIRC	Kidney renal clear cell carcinoma	531	72	***	523	100	*
KIRP	Kidney renal papillary cell carcinoma	289	32	***	286	60	*
LAML	Acute myeloid leukemia	151	–	–	173	70	–
LGG	Brain lower grade glioma	525	–	–	518	207	–
LIHC	Liver hepatocellular carcinoma	373	50	***	369	160	–
LUAD	Lung adenocarcinoma	515	59	***	483	347	*
LUSC	Lung squamous cell carcinoma	501	49	***	486	338	*
MESO	Mesothelioma	86	–	–	182	3	–
OV	Ovarian serous cystadenocarcinoma	379	–	–	426	88	*
PAAD	Pancreatic adenocarcinoma	178	4	*	179	171	*
PCPG	Pheochromocytoma and paraganglioma	183	3	–	182	3	–
PRAD	Prostate adenocarcinoma	496	52	***	492	152	–
READ	Rectum adenocarcinoma	167	10	*	92	318	*
SARC	Sarcoma	263	2	–	262	2	–
SKCM	Skin cutaneous melanoma	471	1	–	461	558	*
STAD	Stomach adenocarcinoma	375	32	***	408	211	*
TGCT	Testicular germ cell tumor	156	–	–	137	165	*
THCA	Thyroid carcinoma	510	58	***	512	337	*
THYM	Thymoma	119	2	–	118	339	*
UCEC	Uterine corpus endometrial carcinoma	544	35	***	174	91	*
UCS	Uterine carcinosarcoma	56	–	–	57	78	*
UVM	Uveal melanoma	80	–	–	–	–	–

*p < 0.05, **p < 0.01, and ***p < 0.001.

### 2.2 Clinical data analyses

The correlation analyses between IL4I1 and different clinical information, including WHO grade, age, survival status, histology, IDH mutation, and 1p/19q codeletion, were performed by utilizing R-packages “limma” and “ggpubr”.

### 2.3 Prognostic analyses

The Kaplan–Meier and log-rank test were used to explore the correlation between mRNA expression of IL4I1 and the prognosis. Moreover, univariate and multiple Cox regression analyses as well as ROC analysis were performed to explore the association between IL4I1 expression and survival outcomes. The findings were displayed as forest plots (using the “forest plot” and “survival” package) and Kaplan–Meier curves (using “survminer” and “survival” packages). Overall survival (OS), disease-specific survival (DSS), disease-free interval (DFI), and progression-free interval (PFI) were analyzed using *p* < 0.05. The “pROC” package was used to construct a sensitivity–specificity curve, with the area under receiver operating characteristic (ROC) curve (AUC) indicating the precision of IL4I1 in glioma assessment (30).

### 2.4 Pan-cancer analyses of IL4I1

Analyses of the pan-cancer dataset were carried out using R 4.1.2. The R package “ggpubr” was applied to generate box plots. For some tumors with the limitations of the normal tissue size of the TCGA database (e.g., ACC, CESC, LGG, GBM, etc.), the “Expression Analysis-Box Plots” module in GEPIA2 with data from TCGA and the GTEx portal (http://gepia2.cancer-pku.cn/#analysis) was utilized to compare the differences of IL4I1 expression among tumor and normal tissues (31). Additionally, the UALCAN portal (https://ualcan.path.uab.edu/) and the Human Protein Atlas (HPA) (https://www.proteinatlas.org/) were used for differences analyses of IL4I1 protein between tumor and normal tissues (32, 33).

### 2.5 DNA methylation analyses

Alterations in the DNA methylation of IL4I1 were explored. The MethSurv database (https://biit.cs.ut.ee/methsurv/#) was adapted to examine methylation levels and the prognostic importance of individual CpG of IL4I1 across various grades of glioma (34).

### 2.6 Enrichment analyses

The CGGA dataset was used to analyze differentially expressed genes (DEGs) on the median expression of IL4I1. DEGs were examined through R packages “limma” and “pheatmap”, using *p* < 0.05 and |logFC | ≥ 1. The Gene Ontology (GO) database (https://geneontology.org/) and Kyoto Encyclopedia of Genes (KEGG) database (www.genome.jp/) were performed to investigate DEGs with enriched functions and signaling pathways, respectively, when linking IL4I1 to glioma. Gene set enrichment analysis (GSEA) was applied to investigate the mechanisms of IL4I1. Results were visualized in R-packages “limma,” “org.Hs.eg.db,” “clusterProfiler,” and “enrichplot”.

### 2.7 Single-cell analysis of IL4I1

The TISCH2 database [TISCH (comp-genomics.org)] was applied for single-cell analysis (35). TISCH2 is an scRNA-seq database that emphasizes the TME specifically. It offers abundant cell subtype annotation at the single-cell level. IL4I1 expression in glioma was visualized using heatmaps.

### 2.8 Infiltration of immune cells

The association between IL4I1 levels and immune cell infiltration was evaluated using the TIMER 2 database (http://timer.cistrome.org/) (36). The algorithms of TIMER, EPIC, and XCELL were applied to estimate the number of macrophages. We acquired *p*-values and partial correlation values using Spearman’s rank correlation test adjusted for sample purity. Furthermore, glioma datasets from TCGA and CGGA databases were utilized for Pearson correlation analysis of markers for distinct macrophage subtypes correlated with IL4I1 expression.

### 2.9 Western blotting and quantitative real-time PCR

Equal amounts of protein lysates (20 µg) extracted from cells transfected with NC and small interfering (si) IL4I1 were subjected to SDS-PAGE and transferred to PVDF membranes. Following incubation with 5% skimmed milk for 1 h at room temperature (RT), PVDF membranes were exposed to primary antibodies targeting IL4I1 (1:1,000, ab222102, Abcam), CD11B (1:1,000, ab133357, Abcam), CD204 (1:1,000, ab271070, Abcam), CD206 (1:2000, 60143-1-Ig, Proteintech), CD163 (1:1,000, ab182422, Abcam), CD86 (1:1,000, ab239075, Abcam), and beta-tubulin (1:2000, DF7967, Affinity) overnight. Subsequently, PVDF membranes were incubated with Goat Anti-Rabbit/Mouse IgG (H+L) HRP (1:5,000, Affinity) for 1 h. Chemical imaging was developed using ECL solutions (Epizyme, Shanghai, China). Pictures were obtained using a Bio-Rad imaging system.

The extraction of total RNA from cells transfected with NC and siIL4I1 was done through an RNA Purification Kit (B0004D, EZBioscience, USA). Subsequently, reverse transcription was carried out using RNA (1 μg). After ward, RT-qPCR was done using the SYBR qPCR Master Mix kit (Epizyme, Shanghai). Amplification was detected using QuantStudio (Thermo Scientific, USA). The primer sequences we used are listed in **Table 3**.

**Table 3:** RT-qPCR primers.

Gene name	Primer sequences (5′ to 3′)
Tubulin	Forward: CGAGCCCTACAACTCCATC
	Reverse: CCAATCAGACGGTTCAGGT
IL4I1	Forward: CGCCCGAAGACATCTACCAG
	Reverse: GATATTCCAAGAGCGTGTGCC
CD11B	Forward: GACCTCAGCATCACCTTCA
	Reverse: CCTCACCATCATTTCTCACA
CD204	Forward: AGTGTTTTGTTTTGGGAGAGA
	Reverse: GAGCAGCGATTTCATAGTTGT
CD206	Forward: GGGTTGCTATCACTCTCTATGC
	Reverse: TTTCTTGTCTGTTGCCGTAGTT
CD163	Forward: CTTGGGACTTGGACGATGCT
	Reverse: GCAGGACAATCCCACAAGGA
CD86	Forward: CCATCAGCTTGTCTGTTTCATTCC
	Reverse: GCTGTAATCCAAGGAATGTGGTC

### 2.10 Immunohistochemistry staining

The study protocol was approved (2021809) by the ethics committee of West China Hospital within Sichuan University (Chengdu, China). Glioma and normal brain tissues were collected from West China Hospital and embedded in paraffin. IHC staining was conducted according to methods described previously (37, 38). In brief, sections of thickness 5 µm were utilized for antigen retrieval in Tris-EDTA buffer (pH 9.0) for 15 min at 100°, followed by blockade with 5% BSA for 1 h. Subsequently, sections were exposed to the primary anti-IL4I1 antibody (1:2,000, ab222102, Abcam) overnight. Lastly, sections were treated with the secondary antibody for 1 h at RT, followed by DAB kit, hematoxylin counterstaining, dehydration, and mounting in a medium.

### 2.11 Immunofluorescence staining

Cells (U87, LN229, M0, M1, and M2-like macrophage) were subjected to fixation using 4% paraformaldehyde. Then, they were treated with 0.2% Triton X-100 and 5% BSA. This action was followed by incubation with primary antibodies (IL4I1, 1:50, ab222102, Abcam; CD206, 1:500, 60143-1-Ig, Proteintech). Subsequently, secondary antibodies were applied, and cells were stained with DAPI Fluoromount-G (0100-20, Southern Biotech, USA). IF staining of tumor tissues was carried out in a similar way to that of IHC staining. Following incubation with primary antibodies, sections were treated with Fluor-488 and -594 conjugate secondary antibody (1:1,000, ab150113, ab150080, Abcam) for 1 h and then mounted in DAPI. Pictures were taken by confocal laser scanning microscopy.

### 2.12 Cell culture, macrophage polarization, and siRNA

Human glioma cell lines (U87, LN229) and a human monocytic cell line (THP-1) were purchased from Meisen Cell (Zhejiang, China). U87 and LN229 cells were cultured in DMEM medium. THP-1 cells were cultured in RPMI 1640 medium. Each medium was supplemented with 10% fetal bovine serum (FBS, BIOEXPLORER^®^ Life Sciences, USA) and 1% penicillin–streptomycin. M0 macrophages were acquired by plating THP-1 cells (1 × 10^6^/well) into a six-well plate and incubating them with phorbol myristate acetate phorbol ester (PMA; 100 ng/mL Sigma, USA) for 24 h. For polarization into M1 macrophages, M0 macrophages were incubated with fresh medium containing PMA (100 ng/mL), IFN-γ (20 ng/mL, PeproTech, USA), and LPS (20 ng/mL, Sigma, USA). For polarization into M2-like macrophages, M0 macrophages were cultured with fresh medium containing PMA (100 ng/mL), IL-4 (20 ng/mL, PeproTech, USA), and IL-13 (20 ng/mL, PeproTech, USA). Meanwhile, to transfect siRNA into M0- and M2-like macrophages, 50 nM of NC or siIL4I1 reagent was added to six-well plates using jetPrime (Polyplus, French) according to the manufacturer’s instructions. Cells were cultured at 37°C for 72 h. Then, cells were harvested for WB, RT-qPCR, and IF analyses. siRNAs were synthesized by Youkang Biotechnology (Zhejiang, China) and the sequence was 5′-AAGGCGAUGAAGAAGUUUGAATT-3′.

### 2.13 Preparation of conditioned medium

M0- and M2-like macrophages (2 × 10^6^/well) were seeded in 6-cm culture dishes and incubated with NC or siIL4I1 reagent for 72 h. Subsequently, cell supernatants were centrifuged (500 × *g*, 4°;, 15 min), filtered through a 0.2-μm filter, and stored in aliquots at –80°C. These were collected as conditioned medium (CM) and utilized for co-culture experiments and ELISA.

### 2.14 Enzyme-linked immunosorbent assay

TNF-α, IL-6, TGF-β, and IL-10 in M0- and M2-like macrophages transfected with NC and siIL4I1 were assessed through ELISA kits (Multisciences Biotech).

### 2.15 Assays for wound healing, cell migration, and cell invasion

U87 and LN229 cells (6 × 10^4^/well) were cultivated in 96-well plates in DMEM (100 μL) with 30% CM of M2-macrophages and allowed to grow to 90% confluence. Then, cells were scratched with the IncuCyte (Essen BioScience, USA) system. Images were taken 0 h and 24 h after scratching. For the assay to measure the migration and invasion of glioma cells, Transwell™ 24-well chambers (Corning, USA) were employed as described previously (39). Briefly, M2-like macrophages (2 × 10^5^/well) were cultured in the lower chamber and transfected with NC or siIL4I1 reagent for 72 h. Then, U87 or LN229 cells (2 × 10^4^/well) in serum-free medium were seeded into the upper chamber, which was coated with 50 μL of Matrigel (for the invasion assay) or left uncoated (for the migration assay). After co-culture for 24 h for U87 cells and 48 h for LN229 cells at 37°C, respectively, cells on the lower surface of the chamber were fixed with 4% paraformaldehyde and stained with 0.1% crystal violet. Measurements were evaluated at ≥3 random fields, and each assay was conducted independently thrice.

### 2.16 Assays to measure cell proliferation and EdU staining

U87 and LN229 cells (3 × 10^3^/well) were cultured in 96-well plates with 100 μL of DMEM and 30% CM of M2-like macrophages. The plate was placed in the IncuCyte Live-Cell System, where real-time images were taken every 12 h for 72 h. Subsequently, percent proliferation was visualized based on the phase area confluence using IncuCyte software. In addition, U87 and LN229 cells co-cultured with CM for 72 h were subjected to EdU staining assays using an EdU-488 cell proliferation detection kit (Beyotime, C0071S) following manufacturer protocols.

### 2.17 Statistical analyses

Data were analyzed using R (version 4.1.2) and GraphPad Prism (version 9.4.1). Kaplan–Meier curves and log-rank test were used to assess differences in survival. Univariate and multivariate Cox models identified independent prognostic factors. The significance of differences was tested using one-way analysis of variance or Student’s *t*-tests. Pearson or Spearman coefficient was applied to analyze correlations between variables. Data are the means ± standard error of mean (SEM). Differences were considered significant at *p < 0.05, **p < 0.01, ***p < 0.001, and ****p < 0.0001 for all tests.

## 3 Results

### 3.1 Expression of IL4I1 in pan-cancer

**Figure 1:**
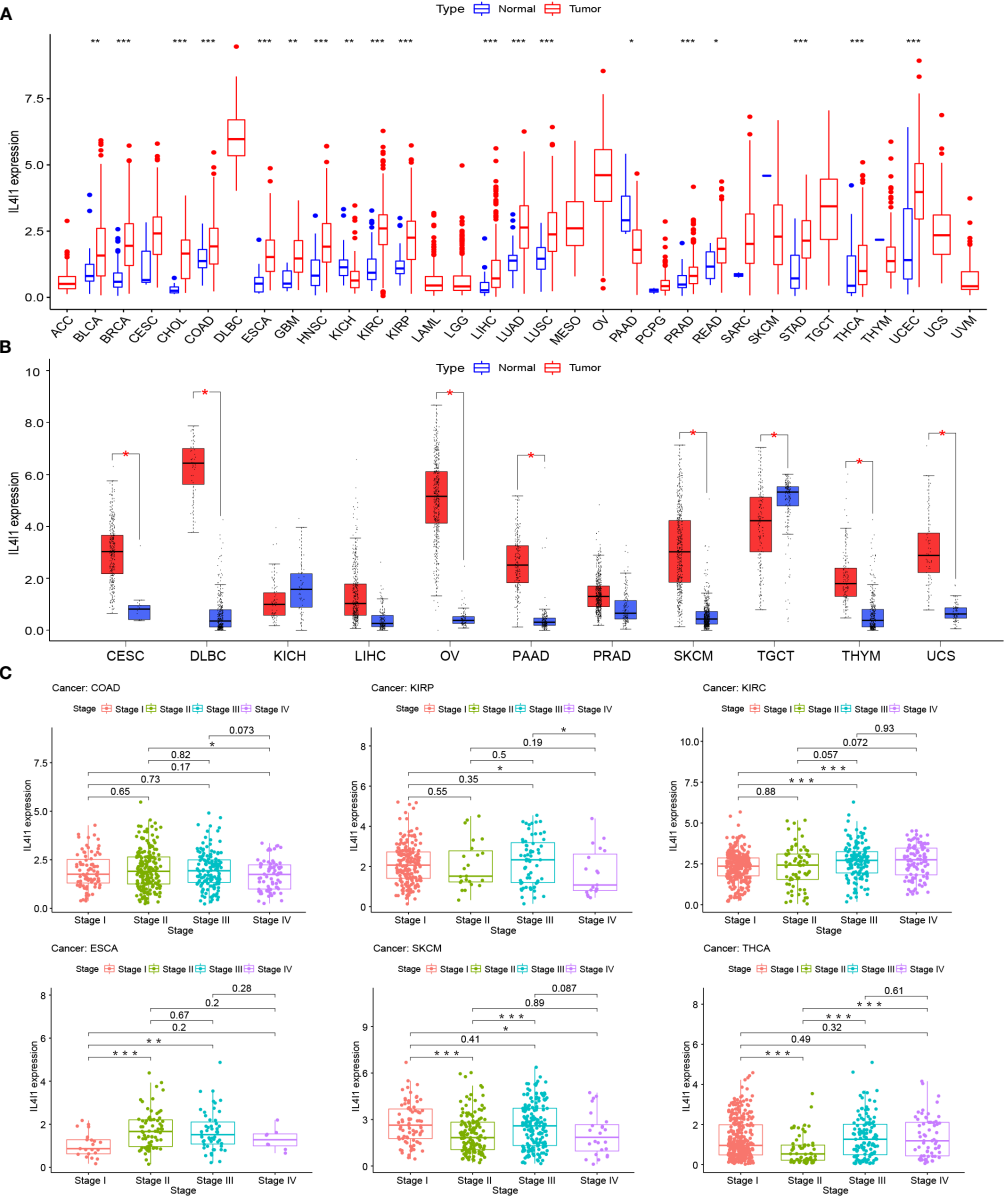
A gene expression analysis of IL4I1 in pan-cancer. **(A)** Expression levels of IL4I1 in the pan-cancer dataset. **(B)** Expression levels of IL4I1 in pan-cancer integrating TCGA and GTEx database. **(C)** Correlation between IL4I1 expression and distinct clinical stages across various tumors. *p < 0.05, **p < 0.01, and ***p < 0.001.

The mRNA expression differences of IL4I1 were explored by combining TCGA and GTEx databases. IL4I1 expression was upregulated in 22 tumor types: BLCA, BRCA, CESC, CHOL, COAD, DLBC, ESCA, GBM, HNSC, KIRC, KIRP, LUSC, LUAD, OV, PAAD, READ, SKCM, STAD, THCA, THYM, UCEC, and UCS. By contrast, it was downregulated in TGCT (**Figures 1A, B**). The abbreviations and detailed information are represented in **Table 2**. Moreover, IL4I1 expression varied significantly in different clinical stages of COAD, KIRP, KIRC, ESCA, SKCM, and THCA, and the main differences were between stage I and III tumors (**Figure 1C**).

Furthermore, we explored protein expression of IL4I1 from the CPTAC dataset and matched the results with IHC images from the HPA database. Results from the CPTAC dataset revealed higher IL4I1 protein in the tissues of HNSC, COAD, BRCA, OV, UCEC, GBM, PAAD, LUAD, and KIRC (*p* < 0.001) (**Supplementary Figures S1C–K**) than that in normal tissues. The findings from the HPA database were consistent with those of the CPTAC dataset. Normal tissues of skeletal muscle, colon, and breast had negative or weak staining of IL4I1 (**Supplementary Figures S1A, B**), and tumor tissues also had weak staining (**Supplementary Figures S1C–E**). Normal ovary, endometrium, cerebral cortex, and pancreas tissues had negative staining of IL4I1 (**Supplementary Figures S1A, B**), whereas OV, UCEC, GBM, and PAAD tissues had strong IL4I1 staining (**Supplementary Figures S1F–I**). Normal lung tissues as well as LUAD, kidney tissues, and KIRC both had strong IL4I1 staining (**Supplementary Figures S1A, B, J, K**).

### 3.2 Prognostic significance of IL4I1 in pan-cancer

**Figure 2:**
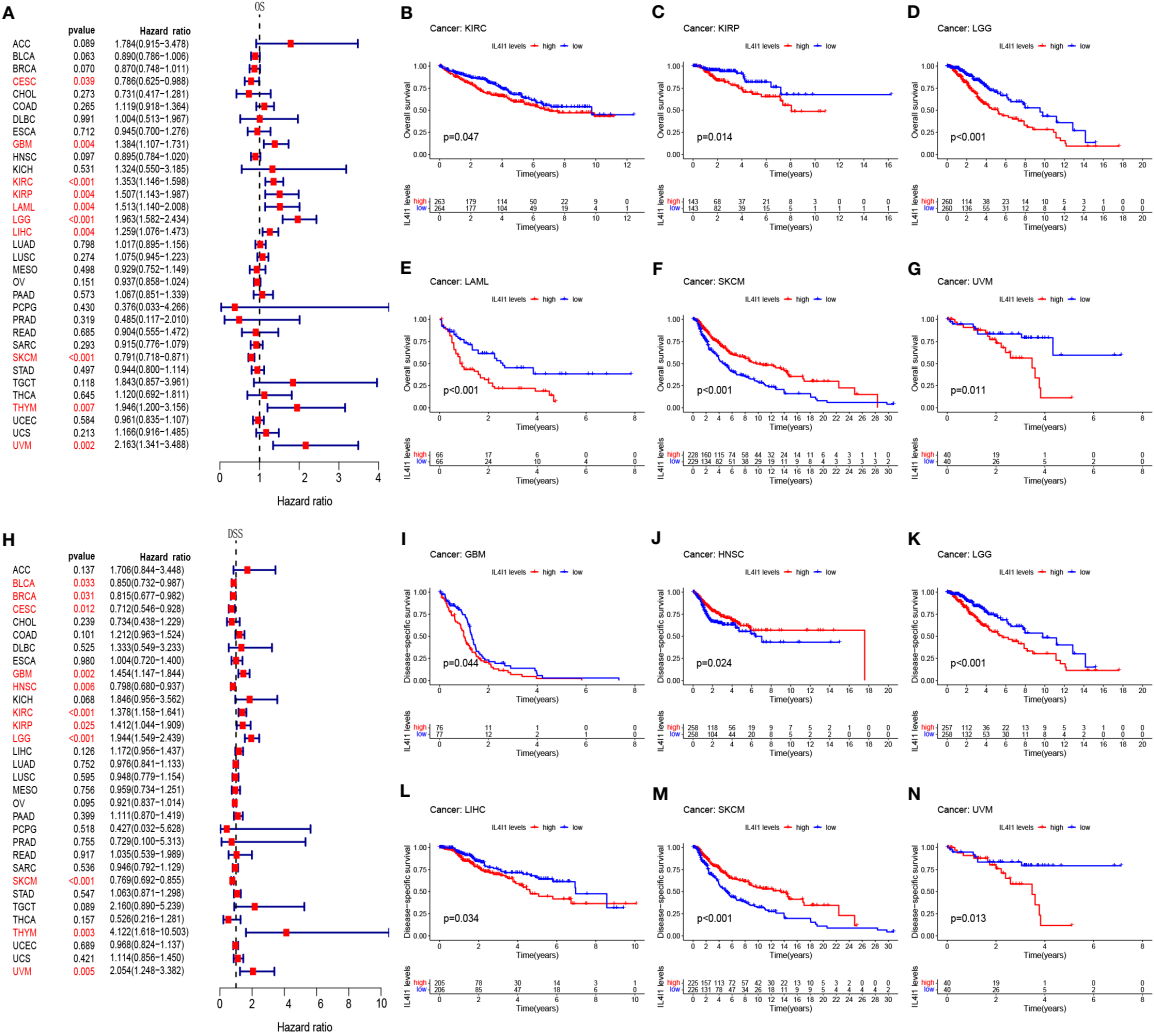
Prognostic correlation between IL4I1 expression and OS and DSS. **(A)** Forest plot of OS in pan-cancer. **(B–G)** Kaplan–Meier curve showing the correlation between IL4I1 expression and OS. **(H)** Forest plot of DSS in pan-cancer. **(I–N)** Kaplan–Meier curve showing the correlation between IL4I1 expression and DSS.

Next, we assessed the prognostic relevance of IL4I1 expression with regard to OS, DSS, DFI, and PFI. Univariate Cox regression analysis revealed that IL4I1 was a risk factor for OS in GBM, LGG, KIRC, KIRP, LAML, LIHC, THYM, and UVM, yet a protective factor in CESC and SKCM (**Figure 2A**). Interestingly, the K-M curve indicated a similar conclusion that heightened IL4I1 expression was correlated with diminished OS in KIRC, KIRP, LGG, LAML, and UVM, but longer OS in SKCM (**Figures 2B–G**). Subsequent analyses using Cox regression for DSS indicated that IL4I1 was a detrimental factor in GBM, LGG, KIRC, KIRP, THYM, and UVM, but a favorable gene in BLCA, BRCA, CESC, HNSC, and SKCM (**Figure 2H**). Likewise, the K-M curves illustrated that elevated IL4I1 level was involved in an unfavorable prognosis in GBM, LGG, LIHC, and UVM, but a better outcome in HNSC and SKCM (**Figures 2I–N**). Furthermore, the forest plot of PFI confirmed the risky role of IL4I1 in GBM, KIRC, KIRP, LGG, PRAD, and THYM, and its role as a protective factor in CESC, HNSC, OV, and SKCM (**Supplementary Figure S2A**), and the K-M curves identified that upregulated IL4I1 was also linked to a poor outcome in GBM, LGG, and THCA, but a better outcome in CESC and SKCM (**Supplementary Figures S2B–F**). Moreover, the results of DFI found that IL4I1 functioned as a risk factor in KIRP patients, but acted protectively in OV (**Supplementary Figure S2G**). Additionally, the K-M curves presented that elevated IL4I1 expression had adverse outcomes in patients with ESCA, but a contrasting result was shown in ACC and CESC (**Supplementary Figures S2H–J**).

Our findings unveil a fundamental correlation of IL4I1 with unfavorable prognoses in GBM, LGG, KIRC, KIRP, LAML, THYM, LIHC, and UVM. In the following study, we delve deeper to comprehensively investigate the importance of IL4I1 expression, with a specific focus on gliomas.

### 3.3 High expression of IL4I1 infers an unfavorable prognosis for glioma patients

**Figure 3:**
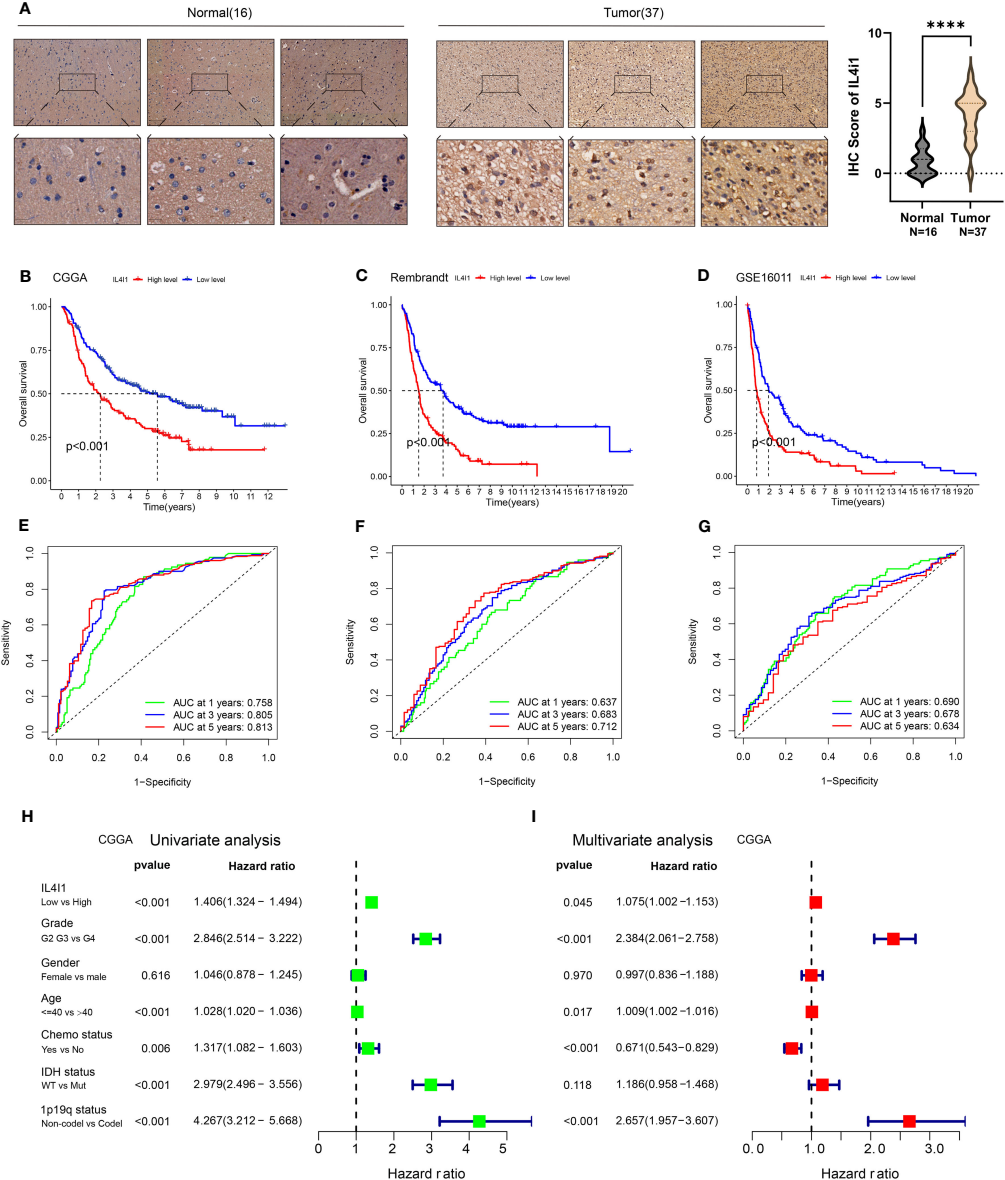
Upregulation of IL4I1 infers an unfavorable prognosis for glioma. **(A)** Representative images (left) and quantification (right) showing IL4I1 staining of normal brain tissues (*n* = 16) and glioma tissues (*n* = 37) with the anti-IL4I1 antibody, with scale bars 100 µm (up) and 20 µm (down). **(B–D)** Kaplan–Meier analysis for OS based on three glioma datasets (CGGA, Rembrandt, and GSE16011). **(E–G)** ROC curves of OS in CGGA, Rembrandt, and GSE16011 datasets. **(H, I)** Univariate and multivariate analyses of OS based on CGGA-glioma datasets. ****p < 0.0001.

The above results from TCGA and GTEx databases have demonstrated higher mRNA and protein expression of IL4I1 in glioma tissues than that in normal brain tissues. To test the accuracy of these results, we applied IHC staining to assess IL4I1 levels in patient-derived samples from West China Hospital. Our IHC results were consistent with the data from TCGA and GTEx databases, demonstrating enrichment of IL4I1 in glioma tissues relative to that in normal tissues (**Figure 3A**). Furthermore, TCGA results indicated a connection between IL4I1 expression and a dismal prognosis in glioma patients. To confirm these data, we investigated the prognostic significance of IL4I1 in three additional independent glioma datasets (**Table 1**). The K-M curves suggested that patients with high expression of IL4I1 had notably unfavorable prognoses relative to the low-expression group (*p* < 0.001, **Figures 3B–D**). The corresponding AUC values for 1-, 3-, and 5-year survival were 0.758, 0.805, and 0.813 in the CGGA dataset (**Figure 3E**); 0.673, 0.683, and 0.712 in the Rembrandt dataset (**Figure 3F**); and 0.690, 0.678, and 0.634 in the GSE16011 dataset (**Figure 3G**). Moreover, univariate Cox regression analysis of the CGGA dataset (**Figure 3H**) showed that IL4I1 level was an independent factor (low vs. high, *p* < 0.001) for predicting glioma patients’ prognosis. Multiple Cox regression results (**Figure 3I**) confirmed that IL4I1 was an independent variable (low vs. high, *p* < 0.045) affecting patient outcomes, even after adjustment for grade, gender, age, chemotherapy status, IDH, and 1p19q status.

These results substantiate the correlation between IL4I1 expression and the prognosis of glioma patients, which demonstrate the potential of using IL4I1 as a reliable prognostic indicator for glioma patients.

### 3.4 High expression of IL4I1 implicates malignant progression of glioma

**Figure 4:**
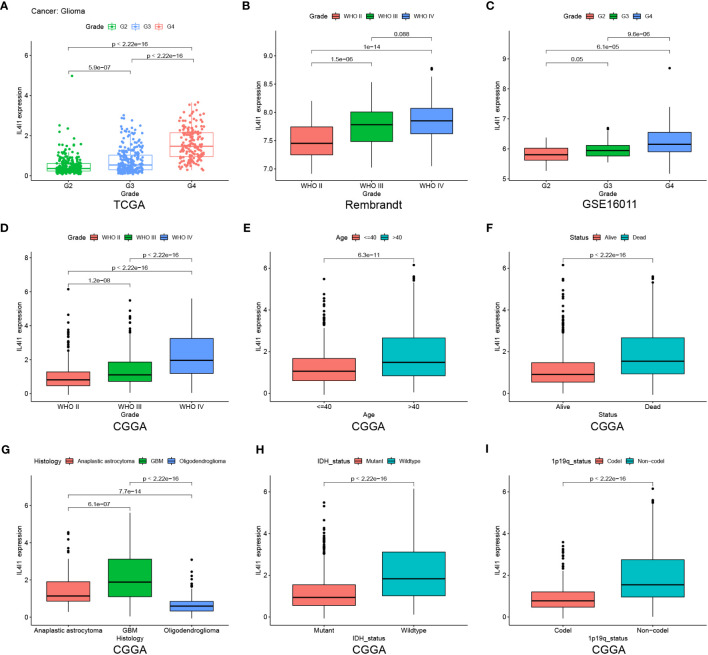
Heightened IL4I1 expression is correlated with the malignant phenotype of gliomas. **(A–D)** IL4I1 shows a significant increase in GBM (WHO IV) in TCGA, Rembrandt, GSE16011, and CGGA datasets. Additionally, box plots visualizing the associations between IL4I1 and various clinical characteristics based on the CGGA dataset. Specifically: **(E)** Age. **(F)** Status. **(G)** Histology. **(H, I)** High expression of IL4I1 in IDH wild-type and 1p/19q non-codel gliomas.

After elucidating the prognostic relevance of IL4I1, we further investigated the correlation between IL4I1 and tumor progression in glioma. Analyses based on data from TCGA, Rembrandt, GSE16011, and CGGA datasets consistently indicated a noticeable increase in IL4I1 levels with higher tumor grades, particularly in WHO grade IV compared with grades II and III (**Figures 4A–D**). In addition, we examined the association between IL4I1 expression and clinical characters in glioma using the CGGA dataset (**Table 4**). Results indicated a significant upregulation of IL4I1 in individuals over the age of 40 years (**Figure 4E**) and markedly higher levels in deceased individuals compared with those who were alive (**Figure 4F**). Furthermore, we observed that IL4I1 was increased substantially in a glioblastoma (WHO IV) compared with an oligodendroglioma (WHO II) and anaplastic astrocytoma subtypes (WHO III) (**Figure 4G**). Moreover, increased IL4I1 was evident in patients with a wild-type IDH mutation (**Figure 4H**) and non-co-deletion of 1p/19q status (**Figure 4I**), both of which are well-established markers associated with worse survival outcomes.

**Table 4:** Clinical information of glioma patients in the CGGA dataset according to IL4I1 expression levels.

Variable	*N*	Overall	High	Low	*p*-value
		*N* = 1018	*N* = 509	*N* = 509	
**Age**	1,017				****
>40		584	333	251	
≤40		433	175	258	
**Survival status**	979				****
Alive		362	118	244	
Dead		617	375	242	
**Grade**	1,013				****
II		291	77	214	
III		334	147	187	
IV		388	282	106	
**Histology**	438				****
Anaplastic astrocytoma		120	54	66	
GBM		225	159	66	
Oligodendroglioma		93	11	82	
**IDH_status**	966				****
Mut		531	184	347	
WT		435	292	143	
**1p19q_status**	940				****
Codel		212	49	163	
Non-codel		728	446	282	

****p < 0.0001.

In summary, IL4I1 expression exhibits an escalating pattern with glioma progression, suggesting its potential involvement in the malignant progression of glioma.

### 3.5 DNA methylation of IL4I1 in glioma

We analyzed differentially methylated CpG islands located in the promoter regions of IL4I1 in glioma using the MethSurv database. The results indicated that five of seven methylated CpGs of IL4I1 (cg06388099, cg05934682, cg03166835, cg24160312, and cg10805880) exhibited significant reductions with increasing tumor grade, and were lower in WHO III compared with WHO II (**Supplementary Figure S3A**). Furthermore, K-M analysis showed that compared with patients with IL4I1 hypermethylation, hypomethylation experienced a worse prognosis in glioma patients (**Supplementary Figure S3B**). These results indicate that DNA methylation of IL4I1 may also play a part in regulating IL4I1 expression and intervening in the prognosis of glioma patients.

### 3.6 IL4I1 is linked to immune functions in glioma

**Figure 5:**
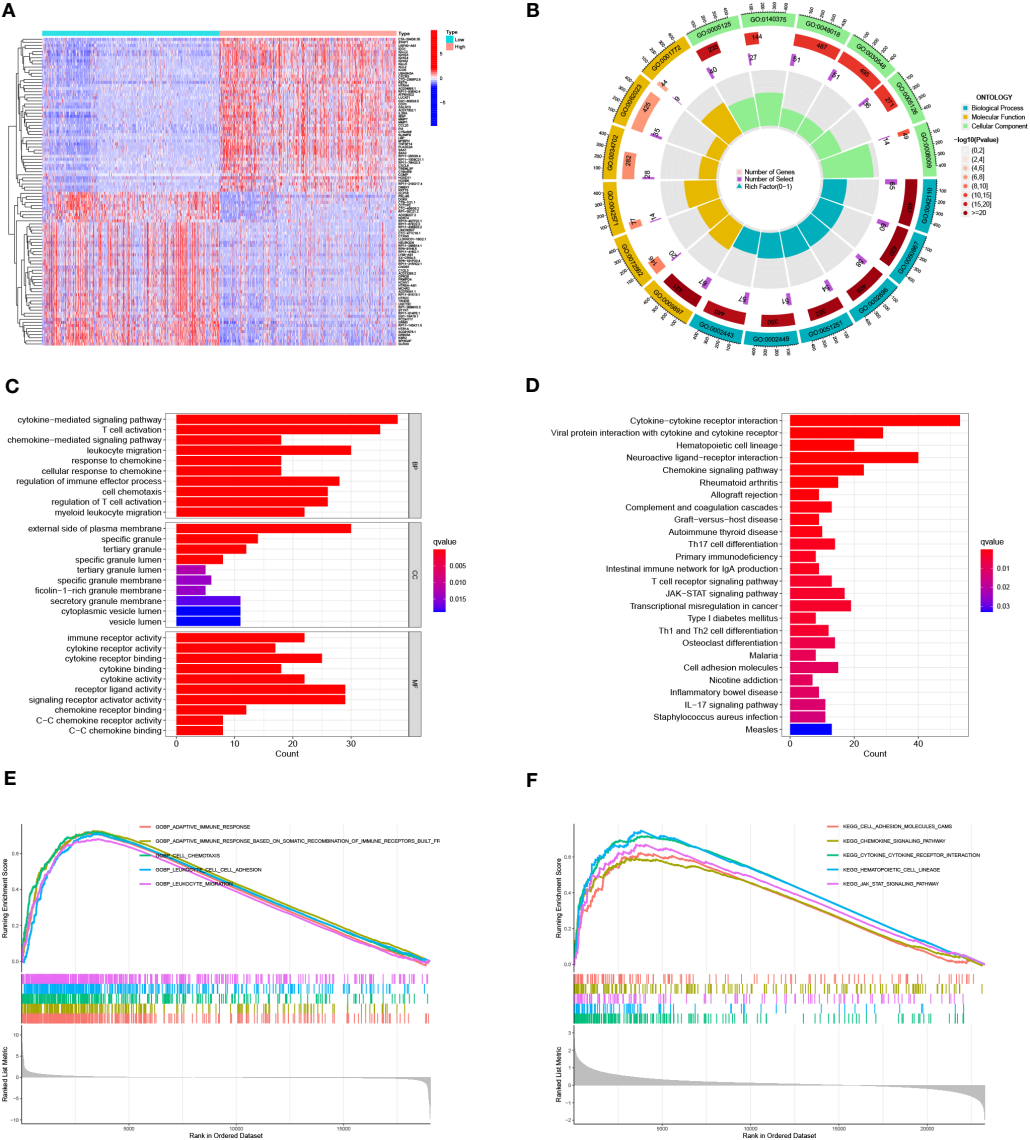
Identification and enrichment analysis of IL4I1 in glioma. **(A, B)** Heat maps showing DEGs that were either upregulated or downregulated. **(C)** GO analysis. **(D)** KEGG analysis of DEGs. **(E, F)** GSEA focusing on functional pathways regarding the IL4I1 expression levels.

In order to explain the biological functions of IL4I1 in glioma, we conducted an analysis of DEGs from the CGGA dataset. Heatmaps in **Figures 5A**, **B** depicted the top 50 DEGs correlated with IL4I1. Within the biological processes of GO terms, most DEGs were enriched in “leukocyte migration”, “cytokine-mediated signaling pathway”, and “regulation of immune effector processes” (**Figure 5C**). KEGG analysis revealed significant enrichment in pathways related to “cytokine–cytokine receptor interaction” and “chemokine signaling” (**Figure 5D**). Correspondingly, the results from GSEA highlighted enrichment in “adaptive immune response” and “leukocyte migration” (**Figures 5E, F**). Moreover, leveraging the CGGA dataset, we further explored the relation between IL4I1 expression and ICs. Findings suggested a positive relation between IL4I1 expression and most ICs (**Supplementary Figures S4A, B**), such as PDCD1, CTLA4, CD274, TGFB1, LAG3, and IDO1 (**Supplementary Figures S4C–H**). These outcomes underscore the critical role of IL4I1 in immune-related functions in glioma.

### 3.7 IL4I1 is notably expressed in M2-like macrophages in glioma

**Figure 6:**
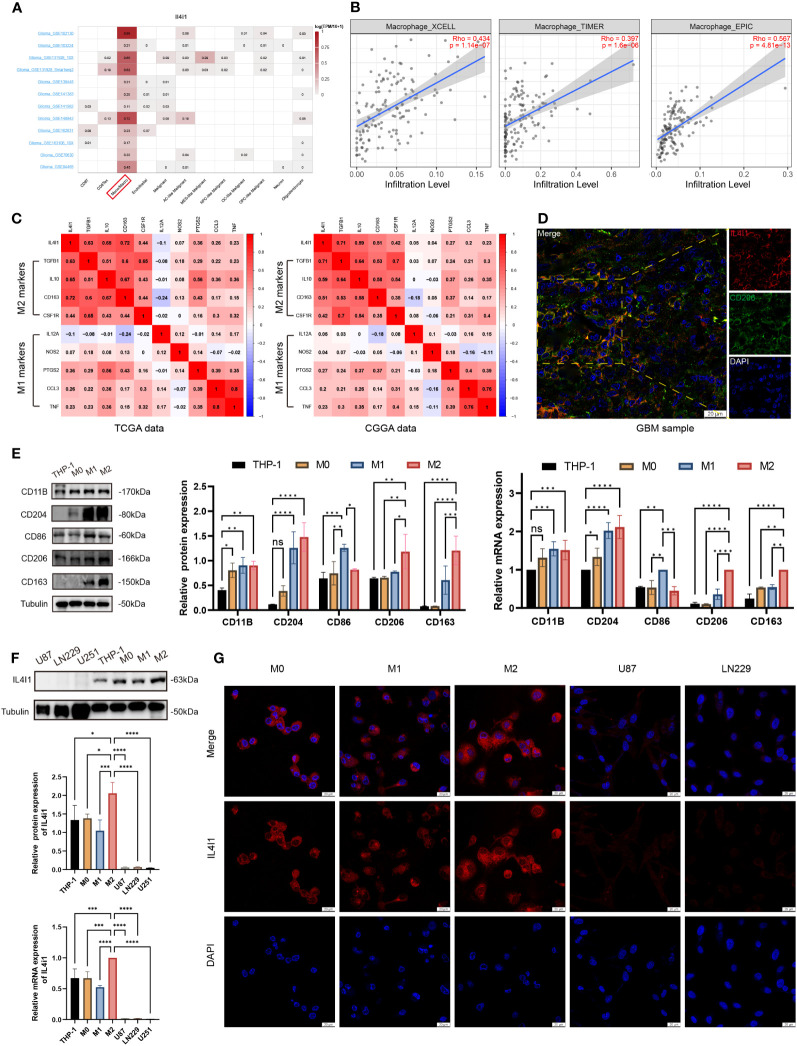
IL4I1 is expressed in M2-like macrophages in glioma. **(A)** Summary of IL4I1 expression in 12 distinct single-cell datasets from glioma patients. **(B)** Association between IL4I1 and macrophages on XCELL, TIMER, and EPIC algorithms. **(C)** Association between IL4I1 expression and markers of M1 and M2 macrophages in CGGA and TCGA databases. Color depth and digital scale represent the strength of association. **(D)** Representative colocalization images from IF staining between IL4I1 and CD206 in clinical glioma specimens. DAPI (blue), IL4I1 (red) and CD206 (green). Scale bar: 20 μm. **(E)** Measurement of protein and mRNA expression levels for markers (CD11B, CD204, CD86, CD206, and CD163) of THP-1, M0, M1, and M2 macrophages using WB and RT-qPCR. **(F)** Assessment of IL4I1 expression in various macrophage subtypes (M0, M1, and M2) and glioma cells (U87, LN229, and U251). **(G)** Representative IF pictures of the difference of IL4I1 among M0, M1, and M2 macrophages, U87, and LN229 cells with DAPI (blue) and IL4I1 (red). *p < 0.05, **p < 0.01, ***p < 0.001, and ****p < 0.0001.

.All aforementioned findings collectively suggested that IL4I1 may function as an independent influencing factor for an unfavorable prognosis, malignant progression, and immune functions in glioma. To delve into the underlying role of IL4I1 in glioma, we assessed its expression across various cell types within the glioma microenvironment, including immune cells, stromal cells, and malignant cells. Results of single-cell analysis highlighted that IL4I1 was expressed predominantly in macrophages (**Figure 6A**), and there was a positive correlation between IL4I1 and macrophages in glioma as indicated by TIMER, XCELL, and EPIC algorithms (**Figure 6B**). Further analyses focusing on the classical phenotype markers of macrophages revealed that IL4I1 exhibited a positive association with markers for M2 (TGFB1, IL10, CD163, and CSF1R), while displaying an inconsistent association with M1 markers (IL12A, NOS2, PTGS2, CCL3, and TNF) based on TCGA-GBM and CGGA cohort data (**Figure 6C**). Furthermore, IF staining targeting CD206 (prominent marker of M2-like macrophages), in conjunction with IL4I1 reaffirmed substantial co-expression of IL4I1 with TAMs in clinical glioma specimens (**Figure 6D**). Subsequently, we validated IL4I1 expression *in vitro*. As expected, THP-1 cells differentiated successfully into M0, M1, and M2 macrophage as the results of WB and RT-qPCR (**Figure 6E**). Expression of the macrophage markers CD11B and CD204 was upregulated significantly across M0-, M1-, and M2-like macrophages. Specifically, CD86 (marker of M1 macrophages) exhibited preferential upregulation in the M1 population, whereas CD206 and CD163 (characteristic of M2 macrophages) displayed greater expression in the M2 population. Subsequent assessment of IL4I1 expression in various macrophage subtypes (M0, M1, and M2) and glioma cells (U87, LN229, and U251) revealed that IL4I1 was expressed primarily in macrophages, particularly in the M2 subtype (**Figure 6F**). Intriguingly, IL4I1 expression was lower in M1-like macrophages compared with that in M0 macrophages. Notably, IL4I1 expression was significantly lower in glioma cells (U87, LN229, and U251). This consistent pattern was also confirmed through IF staining, underscoring the upregulation of IL4I1 in M2-like macrophages (**Figure 6G**).

In a word, our findings indicate that M2-like macrophages in the TME of glioma exhibit notably substantial expression of IL4I1.

### 3.8 IL4I1 in M2-like macrophages induces the migration and invasion of co-cultured glioma cells

**Figure 7:**
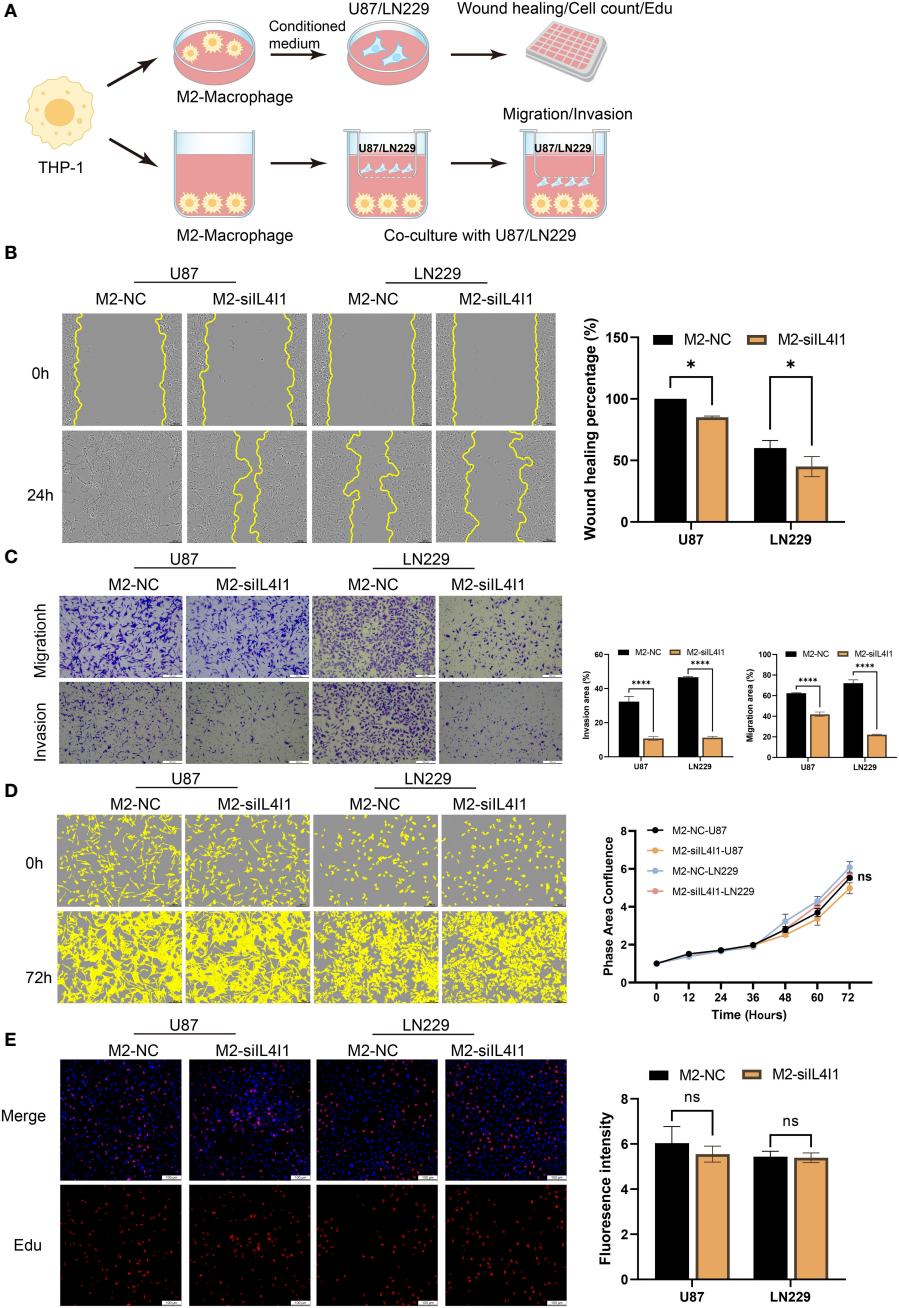
The influence of IL4I1 in M2-like macrophages on proliferation, migration, and invasion of co-cultured glioma cells. **(A)** A schematic representation of the co-cultured model illustrating the M2-like macrophage effects on glioma cells under NC and siIL4I1 conditions. **(B, D, E)** The conditional medium obtained from M2-like macrophage was utilized to incubate with glioma cells (U87 and LN229) for 24 h or 72 h. The effects of IL4I1 in M2-like macrophage on migration and proliferation of U87 and LN229 were evaluated by wound scratch assay **(B)**, IncuCyte **(D)**, and Edu assay **(E)**, respectively. **(C)** Representative pictures and quantification of migration and invasion were obtained through transwell assays following co-cultivation of M2-like macrophages with glioma cells. *p < 0.05 and ****p < 0.0001.

An increasing body of evidence suggests that TAMs can augment tumor occurrence, migration, and invasion of tumor cells (39–41). Therefore, we established a co-culture model to interrogate the effects of IL4I1 in M2-like macrophages on the proliferation, migration, and invasion of glioma cells *in vitro* (**Figure 7A**). In validation experiments, wound-healing assays revealed relatively reduced migration of U87 and LN229 cells upon exposure to the CM from M2-siIL4I1 compared with M2-NC (**Figure 7B**). In addition, stable silencing of IL4I1 expression in M2-like macrophages significantly hampered the migration and invasion of glioma cells, as observed in transwell assays (**Figure 7C**). However, assessments of cell proliferation through EdU staining and IncuCyte Live-Cell assays demonstrated that the CM from M2-siIL4I1 had no impact on proliferation of U87 or LN229 cells (**Figures 7D, E**).

Collectively, our results show that IL4I1 expression in M2-like macrophages plays crucial roles in aggressive progression of human glioma cell lines.

### 3.9 Silencing IL4I1 restrains M2-like macrophages polarization *in vitro*

**Figure 8:**
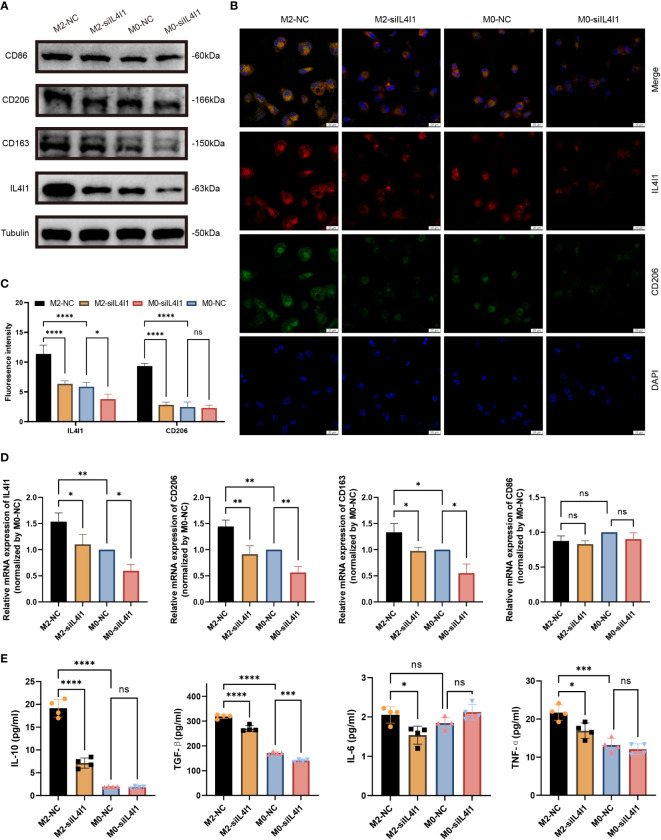
IL4I1 mediates M2-like macrophage polarization. THP-1 cell-derived M0 and M2-like macrophages were transfected with either a negative control (NC) or siIL4I1 siRNA reagent (50 nM) for 48 h. **(A)** The expression levels of IL4I1, CD86, CD163, and CD206 were examined by W-B. **(B, C)** A fluorescence microscope was used to observe the expression of IL4I1 (red) and CD206 (green). **(D)** The expression of IL4I1, CD206, CD163, and CD86 was detected by RT-qPCR. **(E)** ELISA was employed to measure the concentrations of IL-10 and TGF-β (M2-like macrophage markers), as well as the IL-6 and TNF-α (M1-like macrophage markers) in the cell supernatants. *p < 0.05, **p < 0.01, ***p < 0.001, and ****p < 0.0001.

We implemented effective siRNA-mediated silencing of IL4I1 expression in M0- and M2-like macrophages. W-B (**Figure 8A**) and RT-qPCR (**Figure 8D**) results indicated that silencing IL4I1 expression resulted in a decrease in levels of CD206 and CD163 (markers of M2-like macrophages), while having no effect on CD86 (marker of M1 macrophages). IF staining depicted lower levels of the M2 marker, CD206, in IL4I1-silenced M0- and M2-like macrophages compared with siNC controls (**Figures 8B, C**). Moreover, ELISA results showed that si-IL4I1 macrophages expressed notably lower levels of markers of M2 polarization (IL10 and TGFB1) relative to markers of M1 polarization (TNF and IL6) (**Figure 8E**).

In summary, these results demonstrate that IL4I1 induced polarization of M2-like macrophages *in vitro*.

## 4 Discussion

IL4I1 has been associated mainly with the immunoregulation and oncogenic properties of multiple tumors, which could be attributed to its ability to convert amino acids, generate H_2_O_2_, or exhibit an enzyme-independent function (42, 43). Specifically, IL4I1 has been demonstrated to oxidize phenylalanine into phenylpyruvic acid and H_2_O_2_, as well as to convert tyrosine and Trp into hydroxyphenylpyruvic (HPP) acid and I3P, respectively (44, 45). Previous studies have shown that IL4I1 could augment growth vitality of cancer cells and diminish the motility of CD8+ T cells through the I3P-aryl hydrocarbon receptor (AHR) axis (17). Some studies have indicated that IL4I1 could promote the malignant progression of melanoma, KIRC, and LIHC by reprogramming the TME (20, 21, 23). Moreover, the catabolism I3P and H_2_O_2_ of Trp could suppress the activity of cytochrome P450 enzymes, which could explain why AHR activation by IL4I1 is more prominent compared with that by IDO1 or TDO2 (46). In the present study, we demonstrated upregulation of IL4I1 expression across 22 tumor types through pan-cancer analysis, with notably elevated levels in glioma. Furthermore, comprehensive analyses revealed higher expression of IL4I1 protein in tissues of HNSC, COAD, BRCA, OV, UCEC, GBM, PAAD, LUAD, and KIRC compared with that in normal tissues. Survival outcomes from univariate Cox regression analysis indicated a remarkable association between IL4I1 expression and a dismal prognosis across various cancer types, including GBM, LGG, KIRC, KIRP, LAML, THYM, LIHC, and UVM. Consistent with data from previous studies, these findings proved the critical effect of IL4I1 in diverse tumor types, emphasizing the potential of developing IL4I1-targeting inhibitors as a prospective and broad-spectrum avenue for cancer therapy.

The heterogeneity and complexity of epigenetics of glioma hamper treatment and diagnoses. Despite tremendous efforts to reduce recurrence and mortality, this malignancy results in a poor prognosis (1). Herein, we showed a significant upregulation of IL4I1 expression in glioma tissue than that in normal tissue at both transcriptional and protein levels. Our findings also indicated that high expression of IL4I1 elicited a malignant phenotype of glioma. Moreover, IL4I1 expression visibly increased with the tumor grade, showcasing higher levels in WHO grade IV compared with grades II and III. It was also highly enriched in malignancy-associated phenotypes, including the glioblastoma subtype, IDH wild type, and 1p/19q co-deletion. Furthermore, analyses of IL4I1 promoter methylation indicated that methylated CpGs of IL4I1 indeed decreased with tumor grade, and hypomethylation was involved in a poorer prognosis in glioma patients. Taken together, the results suggested that IL4I1 was related to more aggressive biologic processes within glioma as other solid and hematologic malignancies (17, 47, 48). Most likely, these malignant phenotypes could contribute to tumor progression and resistance to therapy, leading to a worse prognosis. Meanwhile, by using Cox regression analysis combined with KM survival analysis in three glioma datasets, we proved that IL4I1 could be regarded as an independent prognostic indicator in glioma. This observation was consistent with that in earlier studies: high IL4I1 expression predicts a worse prognosis in OV (19), HNSC (24), LIHC (48), and KIRC (23).

Within the TME, infiltrating macrophages predominantly exhibit the M2 subtype and are known as TAMs, which contribute critically to promote the proliferation and metastasis of tumor cells (49, 50). The infiltration and activation of TAMs are also well-known factors associated with the prognosis of glioma. For instance, TAM-associated PGK1 phosphorylation has shown correlations with glioma grade, patient prognosis, and tumorigenesis (51). TAM-secreted VEGFA stimulates angiogenesis and supports glioma growth (41). In the present study, we found that IL4I1 was closely involved in immune-related function with TAMs in glioma. Through enrichment analyses, we found that DEGs between IL4I1 subgroups were significantly enriched in immune response, notably in leukocyte migration and cytokine signaling. Meanwhile, correlation analyses also displayed that IL4I1 had a positive correlation with a variety of ICs, such as PDCD1, CTLA4, CD274, TGFB1, LAG3, and IDO1. Furthermore, upon conducting single-cell analysis on gliomas, it was observed that IL4I1 expression was localized predominantly in macrophages. We also identified a positive relation between IL4I1 and macrophages in glioma from the algorithms of TIMER, XCELL, and EPIC. Deeper analysis displayed that IL4I1 was positively correlated with the markers of M2-like macrophages, and IF staining between CD206 and IL4I1 confirmed that IL4I1 was substantially co-expressed with TAMs in clinical glioma specimens. Finally, the results of *in vitro* experiments (WB, RT-qPCR, and IF staining) revealed IL4I1 to be expressed specifically on M2-like macrophages. Collectively, these findings implicate that the immunological role of IL4I1 in glioma might be attributed to the TME where TAMs exhibit heightened IL4I1 expression.

To date, radiotherapy and chemotherapy have been regarded as the most expedient therapies (except surgical excision) for glioma patients (52). Temozolomide, being the only primary-line chemotherapy drug, has displayed notable resistance (53). Recently, while immune checkpoint inhibitors (ICIs) and chimeric antigen receptor T cell (CAR-T) therapy have demonstrated success in treating various solid tumors, the limited lymphocyte responsiveness within the TME has not yielded promising therapeutic outcomes in glioma immunotherapy (54, 55). As a large proportion of cells in the tumor endoplasm, TAMs have been considered as a more promising direction for immunotherapy (10, 12, 51). New therapeutic strategies targeting TAMs have been anchored in interfering with cascade pathways, reconstituting TAMs with drugs or therapeutic genes, and depleting TAMs (12). Previous studies have indicated that IL4I1 could regulate the immune properties of OV (19), KIRC (23), and LIHC (22). Meanwhile, researchers found that the ectopic expression of IL4I1 on glioma cells can promote their migration ability (17). In the present study, we demonstrated that IL4I1 was mainly expressed on M2-like macrophages but not on glioma cells. Furthermore, we constructed a co-culture model to investigate the direct effects of M2-like macrophage-intrinsic IL4I1 in glioma cell response and used CM derived from siIL4I1-treated M2-like macrophages to stimulate glioma cells. We demonstrated that knock down of IL4I1 expression in M2-like macrophages resulted in a reduction in the migration and invasion capabilities of co-cultured glioma cells. Furthermore, IL4I1 induced the polarization of M2-like macrophages *in vitro*. These results suggested that TAMs with increased IL4I1 expression could drive glioma progression and regulate the M2-subtype polarization. This insight undoubtedly offers a more comprehensive and in-depth understanding, building upon previous studies regarding the regulatory role of IL4I1 in glioma progression.

In summary, we demonstrated notable upregulation of IL4I1 expression across various tumor tissues, which correlated with an unfavorable prognosis. IL4I1 exhibited specific overexpression on TAMs and tissues and promoted the progression of malignant gliomas. Moreover, IL4I1 accelerated the polarization of the M2-like macrophages and induced the invasion and migration of co-cultured glioma cells. IL4I1 emerges as a promising immunotherapy target for modulating TAMs selectively and stands as a novel macrophage-related prognostic biomarker in glioma. However, certain limitations within our study exist and will guide our future research directions. Firstly, despite the inclusion of data from human samples and *in vitro* experiments, further investigations necessitate the use of specific IL4I1^CX3CR1KO^ mice models to delve deeper into the IL4I1-mediated downstream mechanism between M2-like macrophages and glioma, encompassing glioma progression and M2-like macrophage polarization. Secondly, because of the limited growth and migration capacity of differentiated M2-like macrophages *in vitro*, we did not obtain significant results regarding the influence of IL4I1 on the proliferation and migration of these cells. Thirdly, owing to the simplification of *in vitro* cell systems, our focus rested primarily on the impact of IL4I1 on M2-like macrophages. However, utilizing IL4I1^CX3CR1KO^ mice models for glioma would allow for more comprehensive exploration of IL4I1 patterns and functions between M2-like macrophages and other immune cell subsets and ICs.

## Data availability statement

The original contributions presented in the study are included in the article/**Supplementary Material**. Further inquiries can be directed to the corresponding authors.

## Ethics statement

The studies involving humans were approved by the ethics committee of West China Hospital within Sichuan University (2021809). The studies were conducted in accordance with the local legislation and institutional requirements. The participants provided their written informed consent to participate in this study. Ethical approval was not required for the studies on animals in accordance with the local legislation and institutional requirements because only commercially available established cell lines were used.

## Author contributions

FY: Writing – original draft. LW: Methodology, Writing – review & editing. YL: Resources, Writing – review & editing. CD: Supervision, Writing – review & editing. LZ: Formal Analysis, Writing – review & editing. JX: Writing – review & editing.

## Supplementary material

Supplementary Figure 1 IL4I1 protein expression is notably increased in tumor tissues compared to normal tissues (left), with corresponding IHC staining on normal (middle) and tumor (right) tissues in various tumors. **(A, B)** A summary of IL4I1 protein between normal and tumor tissue in the HPA database. **(C)** IHC staining of IL4I1 in the skeletal muscle tissue and HNSC in the HPA database. **(D)** Colon tissue and COAD. **(E)** Breast tissue and BRCA. **(F)** Ovary tissue and OV. **(G)** Endometrium tissue and UCEC. **(H)** Cerebral tissue and GBM. **(I)** Pancreas tissue and PAAD. **(J)** Lung tissue and LUAD. **(K)** Kidney tissue and KIRC. ***p < 0.001.

Supplementary Figure 2 Prognostic correlation between IL4I1 expression and PFI, DFI. **(A)** Forest plot of PFI in pan-cancer. **(B-F)** Kaplan-Meier curve showing the correlation between IL4I1 expression and PFI. **(G)** Forest plot of DFI in pan-cancer. **(H-J)** Kaplan-Meier curve showing the correlation between IL4I1 expression and DFI.

Supplementary Figure 3 Differences in DNA methylation patterns and their impact on the prognosis of IL4I1 in patients with glioma. (MethSurv database). **(A)** The methylated CpGs of IL4I1 were notably reduced in grade III compared to grade II among glioma patients. **(B)** Kaplan–Meier curves illustrated a poorer prognosis associated with hypomethylation.

Supplementary Figure 4 Association between IL4I1 and immune checkpoints. **(A, B)** The heat maps between IL4I1 and top 18 checkpoint genes. **(C)** PDCD1. **(D)** CTLA4. **(E)** CD274. **(F)** TGFB1. **(G)** LAG3 and **(H)** IDO1.
