# Supplementary figures and images for: IL4I1 in M2-like macrophage promotes glioma progression and is a promising target for immunotherapy
Source: Front Immunol. 2024 Jan 5;14:1338244. doi: 10.3389/fimmu.2023.1338244 (PMC10799346; doi:10.3389/fimmu.2023.1338244)

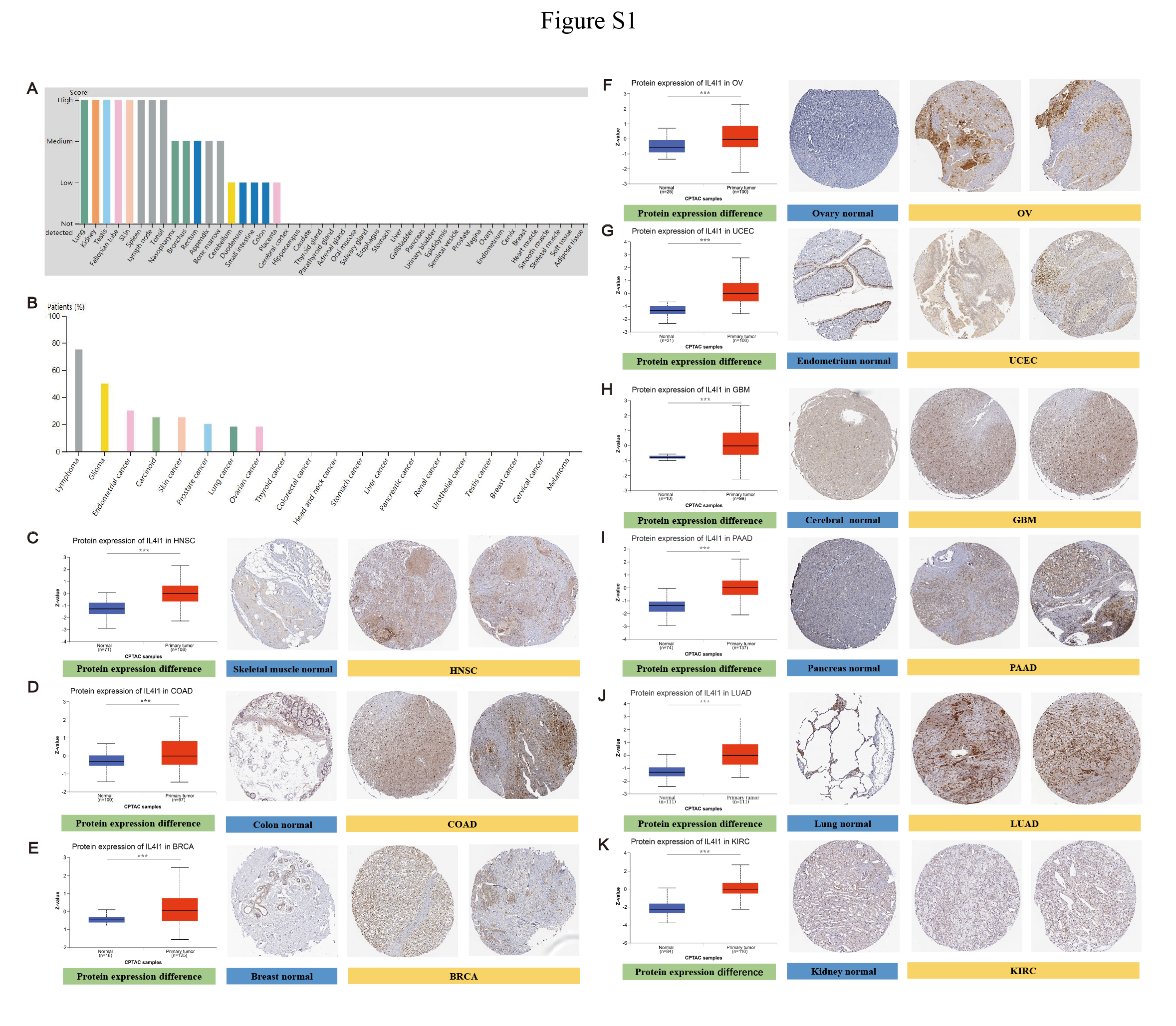

Supplement: Supplementary Figure 1 — IL4I1 protein expression is notably increased in tumor tissues compared to normal tissues (left), with corresponding IHC staining on normal (middle) and tumor (right) tissues in various tumors. (A, B) A summary of IL4I1 protein between normal and tumor tissue in the HPA database. (C) IHC staining of IL4I1 in the skeletal muscle tissue and HNSC in the HPA database. (D) Colon tissue and COAD. (E) Breast tissue and BRCA. (F) Ovary tissue and OV. (G) Endometrium tissue and UCEC. (H) Cerebral tissue and GBM. (I) Pancreas tissue and PAAD. (J) Lung tissue and LUAD. (K) Kidney tissue and KIRC. ***p < 0.001. [file DataSheet_1.zip › Figure S1.tif]

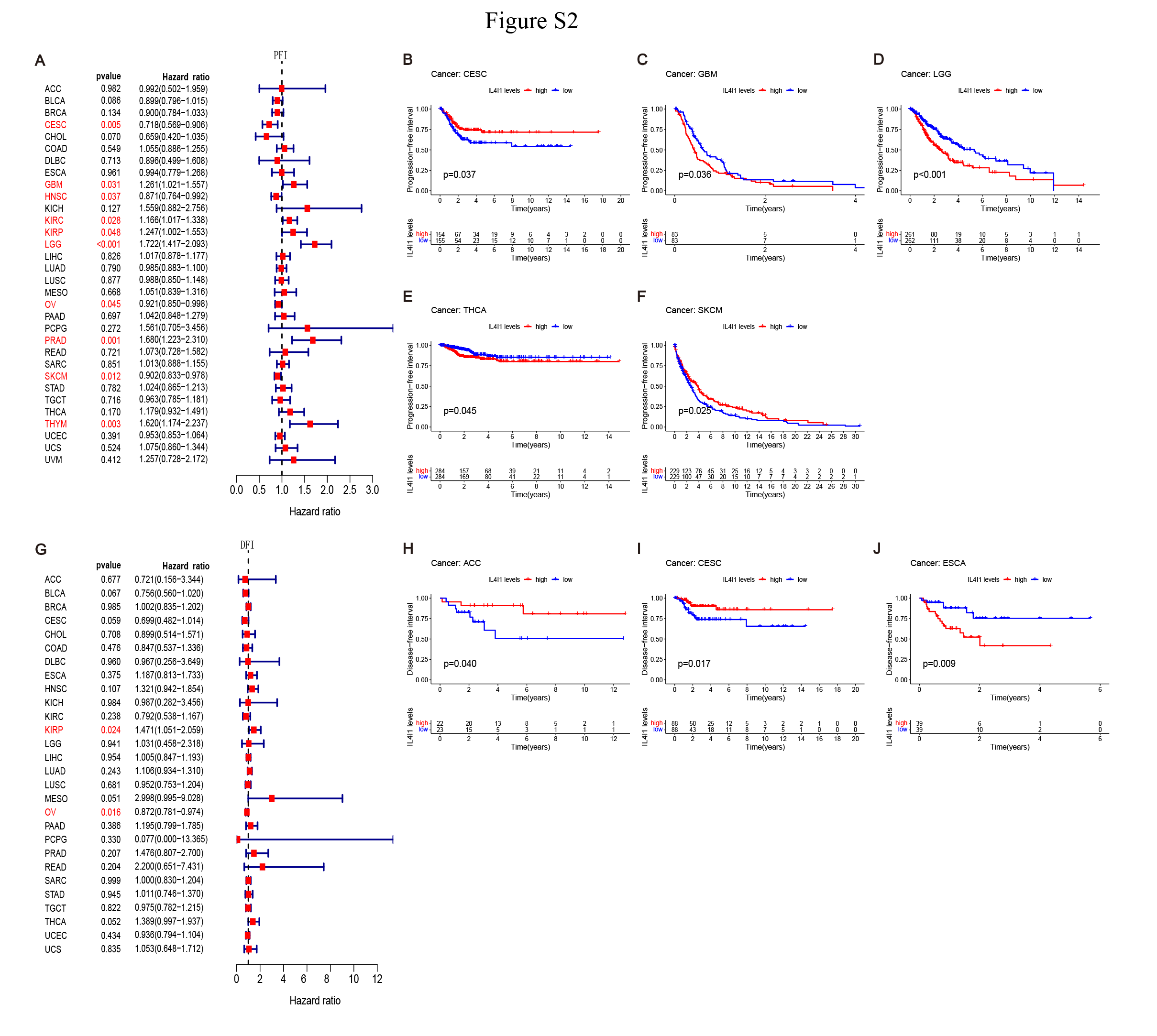

Supplement: Supplementary Figure 1 — IL4I1 protein expression is notably increased in tumor tissues compared to normal tissues (left), with corresponding IHC staining on normal (middle) and tumor (right) tissues in various tumors. (A, B) A summary of IL4I1 protein between normal and tumor tissue in the HPA database. (C) IHC staining of IL4I1 in the skeletal muscle tissue and HNSC in the HPA database. (D) Colon tissue and COAD. (E) Breast tissue and BRCA. (F) Ovary tissue and OV. (G) Endometrium tissue and UCEC. (H) Cerebral tissue and GBM. (I) Pancreas tissue and PAAD. (J) Lung tissue and LUAD. (K) Kidney tissue and KIRC. ***p < 0.001. [file DataSheet_1.zip › Figure S2.tif]

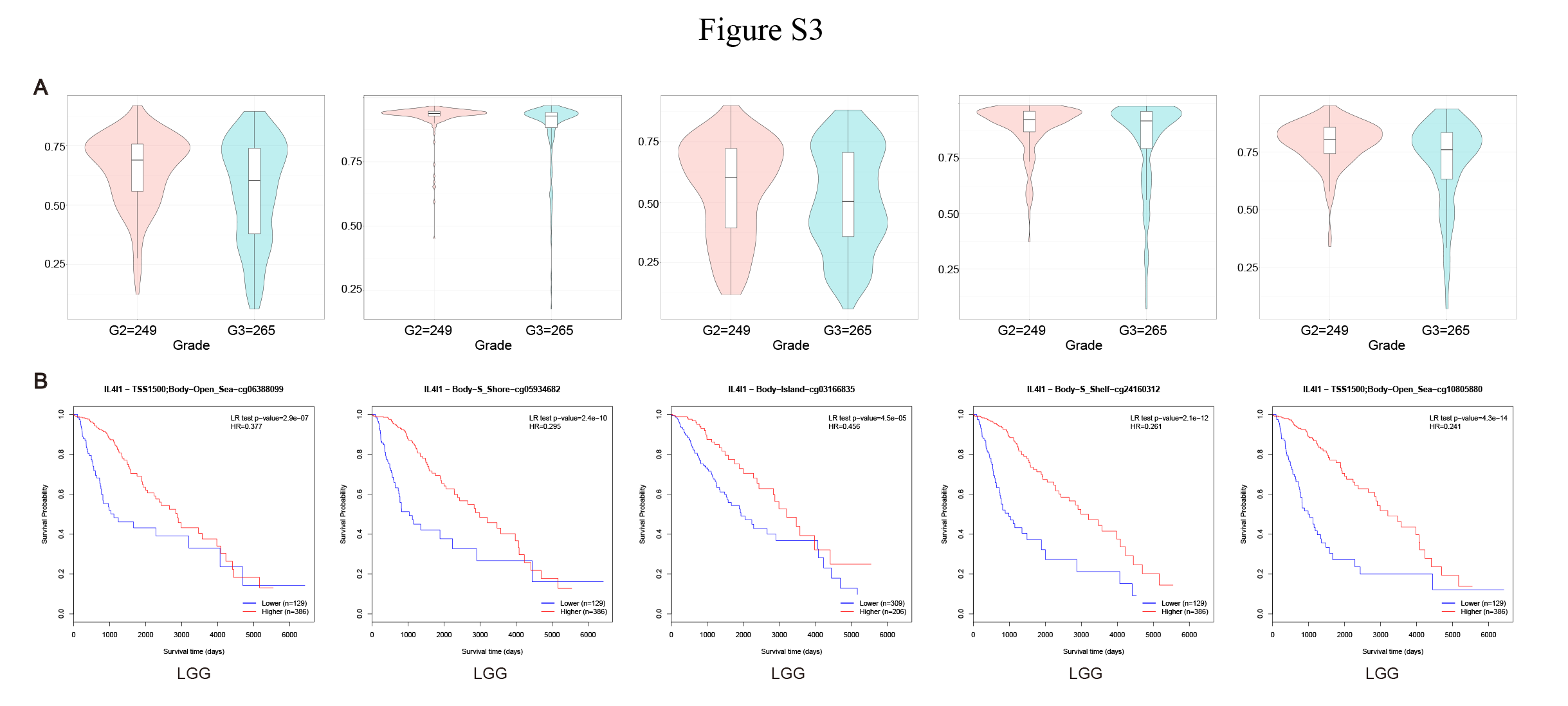

Supplement: Supplementary Figure 1 — IL4I1 protein expression is notably increased in tumor tissues compared to normal tissues (left), with corresponding IHC staining on normal (middle) and tumor (right) tissues in various tumors. (A, B) A summary of IL4I1 protein between normal and tumor tissue in the HPA database. (C) IHC staining of IL4I1 in the skeletal muscle tissue and HNSC in the HPA database. (D) Colon tissue and COAD. (E) Breast tissue and BRCA. (F) Ovary tissue and OV. (G) Endometrium tissue and UCEC. (H) Cerebral tissue and GBM. (I) Pancreas tissue and PAAD. (J) Lung tissue and LUAD. (K) Kidney tissue and KIRC. ***p < 0.001. [file DataSheet_1.zip › Figure S3.tif]

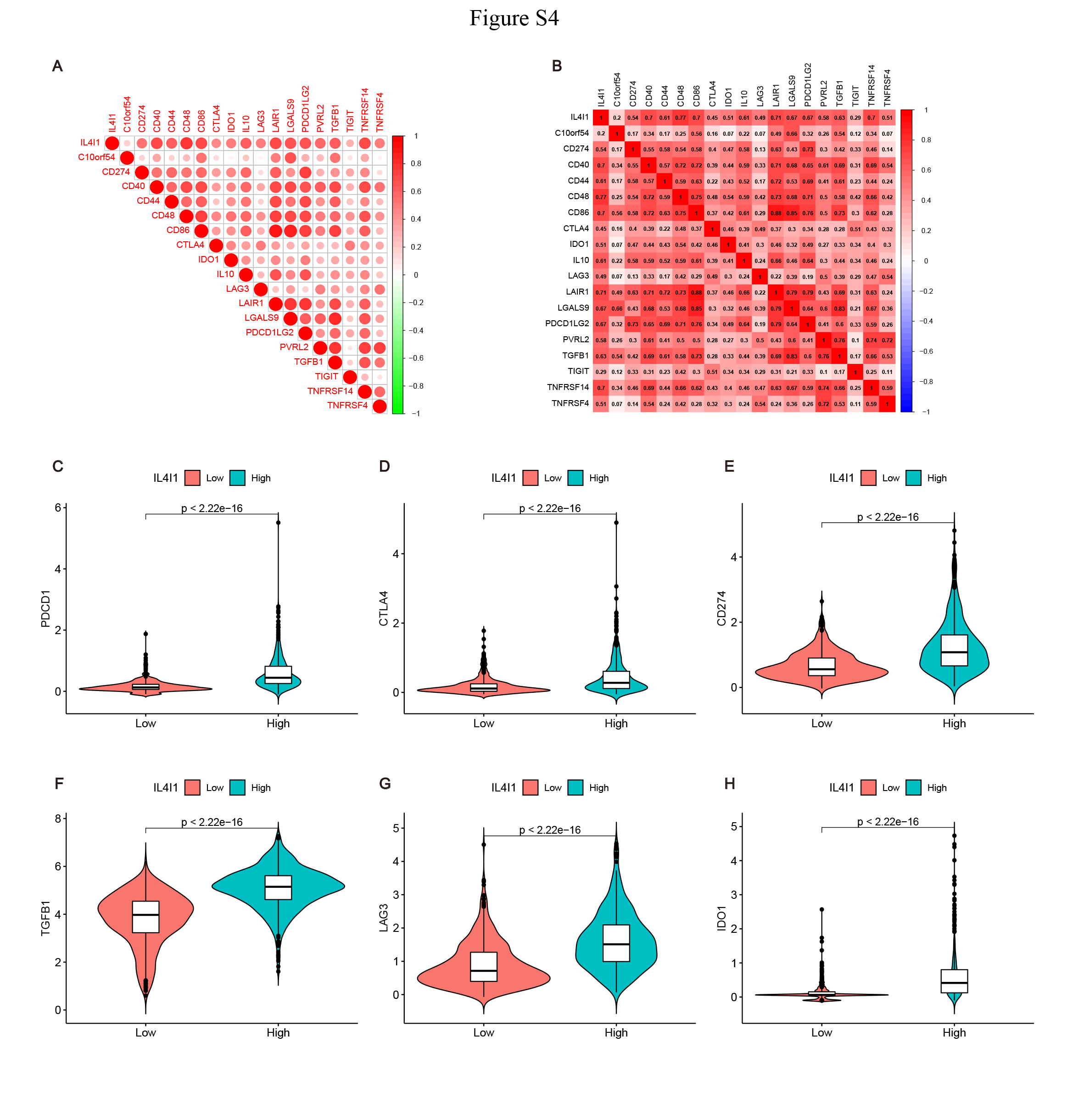

Supplement: Supplementary Figure 1 — IL4I1 protein expression is notably increased in tumor tissues compared to normal tissues (left), with corresponding IHC staining on normal (middle) and tumor (right) tissues in various tumors. (A, B) A summary of IL4I1 protein between normal and tumor tissue in the HPA database. (C) IHC staining of IL4I1 in the skeletal muscle tissue and HNSC in the HPA database. (D) Colon tissue and COAD. (E) Breast tissue and BRCA. (F) Ovary tissue and OV. (G) Endometrium tissue and UCEC. (H) Cerebral tissue and GBM. (I) Pancreas tissue and PAAD. (J) Lung tissue and LUAD. (K) Kidney tissue and KIRC. ***p < 0.001. [file DataSheet_1.zip › Figure S4.tif]
